# Editorial: Modeling the human well-being benefits of ecosystem restoration and management for environmental decision making

**DOI:** 10.3389/fevo.2024.1456660

**Published:** 2024-08-14

**Authors:** Susan H. Yee, Matthew C. Harwell, Joel Hoffman, Tammy Newcomer-Johnson, Marc Russell

**Affiliations:** 1 Gulf Ecosystems Measurement and Modeling Division, Center for Environmental Measurement and Modeling, United States Environmental Protection Agency, Gulf Breeze, FL, United States,; 2 Pacific Ecological Systems Division, Center for Public Health & Environmental Assessment, United States Environmental Protection Agency, Newport, OR, United States,; 3 Great Lakes Toxicology and Ecology Division, Center for Computational Toxicology and Exposure, United States Environmental Protection Agency, Duluth, MN, United States,; 4 Watershed and Ecosystem Characterization Division, Center for Environmental Measurement and Modeling, United States Environmental Protection Agency, Cincinnati, OH, United States,; 5 Center for Computational Toxicology and Exposure, United States Environmental Protection Agency, Gulf Breeze, FL, United States

**Keywords:** ecosystem services, restoration, human well-being, environmental management, natural infrastructure, socio-ecological network

Since the release of the 2005 Millenium Ecosystem Assessment (MEA), there has been ever-growing interest and initiatives to encourage and support the use of natural infrastructure to enhance social and economic benefits to people (White House Council on Environmental Quality et al., 2022). The concept of ‘ecosystem services’ connects changes in ecosystems to the provisioning of goods and services that ultimately convey benefits to human well-being (Millennium Ecosystem Assessment, 2005). However, environmental management decisions are already complex and layering on human well-being benefits can seem intractable because they: require multi-disciplinary socio-ecological information; are saddled with the inherent uncertainty of natural systems; couple science-based information with subjective human values; and are embedded within a decision environment of multiple stakeholder perspectives, multi-objective tradeoffs, and limited resources (Yee et al., 2017).

This Research Topic consists of one review paper, seven original research articles, and two methods papers that present quantitative models, modeling frameworks, and tools to facilitate restoration and management of natural infrastructure to benefit human well-being through delivery of ecosystem services. The contributed papers illustrate how ecosystem services tools can be weaved throughout a generic 6-step decision process (reviewed by Sharpe et al.). These steps include:

1. clarifying the decision context, including potential impacts on stakeholders;
2. defining important objectives of the decision, including characterizing their reliance on underlying ecosystem services, and determining how those objectives will be measured;
3. developing alternative decision options to be considered, including the potential for nature-based solutions to provide ecosystem services;
4. estimating consequences of alternative options on ecosystem services objectives through models, risk analysis, or cost-benefit analysis;
5. evaluating tradeoffs and selecting an option, in consideration of preferences across different stakeholders; and
6. implementing, monitoring, and reviewing outcomes of the decision for ecosystem services and well-being.

Sharpe et al. reviews the use of the National Ecosystem Services Classification System Plus (NESCS Plus) as a unifying terminology, based on the beneficiary-focused concept of final ecosystem goods and services (FEGS), for consistency and compatibility among tools, as decision makers work through a decision-making process. The collection of compatible tools can be used to scope important stakeholders, identify and prioritize ecosystem services objectives, develop measures of ecosystem services for assessment and monitoring, and explore data, maps, and models for comparing decision options.

Hernandez et al. provides an application of FEGS to select objectives for tidal wetland restoration. A quantitative scoping analysis was used during a series of meetings with estuary program managers to identify priority stakeholders, how they benefit from restoration, and the environmental attributes most important to restore. As an alternative when direct engagement may not be practical, Jackson et al. presents a related document-analysis approach to identify ecosystem services benefits of tidal wetland restoration for different regions and organizations across the United States. Ideally, the two approaches can complement each other by providing initial insights on preliminary objectives or key groups based on analysis of existing documents before directly engaging local stakeholders.

De Jesus Crespo et al. applies a socio-ecological network approach to model and map the spatial relationships between flows of one priority ecosystem service objective, avoided sediment delivery to water reservoirs, to end users in Puerto Rico. The study estimates the supply, demand, and vulnerability of sediment retention services in reservoir drainage areas to help identify priorities for watershed-level management of reservoirs.

Singh et al. proposes quantitative methods to assess vulnerability. They propose a probabilistic framework for incorporating disaster risk into ecological risk assessment. Ecosystems and the services they provide may be vulnerable to disasters. Conversely, natural infrastructure may protect against disasters, but evaluating the efficacy of nature-based solutions for hazard risk depends on adequately estimating the likelihood and magnitude of impacts.

A more holistic multi-objective approach for linking actions to well-being benefits is applied by Fulford and Paulukonis, who use principles of network analysis to identify action pathways for achieving social, health, and economic well-being outcomes through changes in ecosystem services, alongside economic and social services. Networks help visualize relationships so that the most influential actions can be identified, and potential trade-offs examined, including selecting actions that do not contradict each other or accomplish redundant outcomes.

Kalaidjian et al. proposes methods for accounting for well-being by first assembling an evidence-base of benefits of natural infrastructure to psychological, social, and physiological human well-being. The study proposes a framework by which well-being objectives can be measured and preferences for natural infrastructure projects compared using utility functions and equity weighted cost-benefit analysis.

Lyon-Mackie et al. applies qualitative and quantitative deliberative methods for assessing the preferences of stakeholders for tradeoffs across benefits of coastal habitat restoration, and explores the degree to which preferences vary geographically. Deliberative processes have advantages over monetary valuation of ecosystem services in that they actively engage stakeholders, promote social learning, lead to shared social values, and provide insights toward implementing habitat restoration efforts that address local values.

Hesley et al. further investigates whether directly involving stakeholders in implementing restoration projects can lead to more successful outcomes, applying a logic model for program evaluation. Community scientists participating directly in coral reef restoration efforts reported behavioral changes, were more confident in communicating and advocating for coral reefs and were more likely to support conservation programs.

The involvement of a community is crucial to revitalization of brownfield sites, as well, especially for addressing community desires for the space. Mastervich et al. evaluated brownfield projects to identify design elements that resulted in ecosystem services benefits. The study also surveyed tools that may be useful for community visioning, identifying potential health and ecosystem services benefits of restoration, prioritizing sites for redevelopment by mapping vulnerabilities and assets, and designing and implementing sustainable projects.

In summary, this Research Topic leverages conceptual models and quantitative approaches to evaluate connections between environmental restoration and management actions, ecosystem condition, ecosystem services, and human health and well-being. Example applications demonstrate transferable approaches to integrate community priorities with nature-based solutions to enhance benefits of environmental remediation, ecological restoration, and community revitalization.
